# Radial Quantitative Ultrasound and Dual Energy X-Ray Absorptiometry: Intermethod Agreement for Bone Status Assessment in Children

**DOI:** 10.1155/2015/232876

**Published:** 2015-04-01

**Authors:** Kar Hau Chong, Bee Koon Poh, Nor Aini Jamil, Nor Azmi Kamaruddin, Paul Deurenberg

**Affiliations:** ^1^Physical Activity and Energy Metabolism Research Group, Faculty of Health Sciences, Universiti Kebangsaan Malaysia, Jalan Raja Muda Abdul Aziz, 50300 Kuala Lumpur, Malaysia; ^2^Department of Medicine, Faculty of Medicine, Universiti Kebangsaan Malaysia, Jalan Yaacob Latif, Bandar Tun Razak, 56000 Cheras, Kuala Lumpur, Malaysia; ^3^Telaga Harbour Marina, Lot 1, 07000 Langkawi, Malaysia

## Abstract

*Aim*. To validate a radial quantitative ultrasound (QUS) system with dual energy X-ray absorptiometry (DXA), a criterion technique in bone status assessment among children. *Methods*. Bone health was evaluated using a radial QUS system (Sunlight Omnisense 8000P) to measure the speed of sound (SOS) at one-third distal radius of the nondominant hand and DXA (Hologic QDR) was used to assess whole body bone mineral density (BMD). *Results*. Some 29.9% of the children were grossly misclassified according to quartiles of BMD and radial SOS. Poor agreement was observed between *Z*-scores of radial SOS and whole-body BMD (mean difference = 0.6 ± 0.9; 95% limits of agreement = −1.4 to 2.6). With a cut-off value of −1.0, radial SOS yielded satisfactory sensitivity (80%) and specificity (93%) for the detection of children with low BMD. *Conclusion*. The observed poor agreement in the present study suggests that radial QUS and DXA are not comparable and hence are not interchangeable in evaluating bone status of the children.

## 1. Introduction

Despite the advances in diagnosis and management, osteoporosis remains a global health problem that causes more than 8.9 million fractures annually worldwide, and its prevalence is expected to further increase due to the ageing population [1, 2]. Although osteoporosis has traditionally been viewed as an “aging-associated” disease, there is evidence that maximizing bone mineral accrual during childhood and adolescence can provide protection against osteoporosis and related fractures later in life [3]. Therefore, it is important to address bone health in younger populations as part of an osteoporosis preventive strategy so that individuals with low bone mass can be identified and suitable intervention programs can be implemented at an early stage.

Several noninvasive densitometric techniques are currently available for bone health monitoring, such as quantitative computed tomography, magnetic resonance imaging, and dual energy X-ray absorptiometry (DXA) [4]. However, they are known to differ in terms of technologies and acquisition methods, making it hard to reach a consensus of which of these techniques provides the best measurement of bone quality and how they are interchangeable. At present, DXA is considered the preferred technique for assessing bone mineral density (BMD) in children and adolescents due to its speed, precision, and robust pediatric reference data [5, 6]. Moreover, there is mounting evidence that DXA-derived bone parameters are related to fracture risks in these growing populations [6]. Nevertheless, DXA is not an ideal technique for serial monitoring of bone status in children due to the issues of radiation exposure, although the doses of radiation are relatively low [7]. Furthermore, the expense of DXA is high and the equipment is neither universally available nor portable for the fieldwork situation [8].

QUS, which employs the technology of ultrasound waves, has gained much popularity in recent years as an alternative technique to evaluate bone status. Compared to DXA and other X-ray based techniques, QUS is more accessible to the general population because of its portability, ease of operation, low cost, and zero-level radiation exposure [9]. In addition, recent reviews of both in vitro and in vivo studies in adults on the clinical performance of QUS suggest that this technology provides information on bone quality in addition to BMD [9, 10], which are equally important in determination of bone status.

A major issue with the widespread use of QUS is that the QUS devices are technologically diverse and differ in terms of measurement sites and bone parameters, with different levels of validating data supporting their applicability in the populations [11, 12]. To date, calcaneus is the only measurement site that has attained acceptable level of scientific validation for the clinical use of QUS in osteoporosis management [11]. However, not all calcaneal devices are applicable to pediatric populations because of inappropriate transducer sizes [13]. Hence, other peripheral skeletal sites such as radius and tibia, which can be measured using handheld probes, have been suggested for the evaluation of bone status in children. Their scientific validity for bone measurements, particularly at distal radius, remains controversial [14–17] and there is limited information available on the usefulness of a radial QUS system as a bone health assessment tool for the healthy child population. Moreover, procedures are not fully standardized for measurements across different QUS devices, which have urged the need of validating each device to ensure its accuracy and precision before introducing it to the population of interest.

Therefore, the present study aimed to validate a radial QUS system for use among children by analysing the agreement between radial QUS parameters and whole-body BMD as measured by DXA as criterion technique.

## 2. Materials and Methods

### 2.1. Study Design and Sampling

This validation study is part of the Nutrition Survey of Malaysian Children, which is a part of the South East Asian Nutrition Surveys (SEANUTS) [18]. SEANUTS is a multicentre study carried out in 16,744 children aged 0.5 to 12 years in Malaysia, Indonesia, Thailand, and Vietnam [19]. A total of 134 children [calculated using G^*^Power version 3.1.3 software [20]: medium effect size: 0.3; power: 95%; level of significance: 5%] aged 7 to 11 years were recruited from four randomly selected national primary schools, each comprised of at least 1000 students, in Kuala Lumpur, Malaysia. Ethical approval was obtained from the Medical Research Ethics Committee of Universiti Kebangsaaan Malaysia (NN-146-2011). Permissions from the Ministry of Education and principals of the schools were also obtained. Written informed consent from parents and verbal assent from the children were obtained before data collection commenced.

### 2.2. Subjects

Included children were all ethnic Malays, aged between 7 and 11 years, and apparently physically and mentally healthy boys and girls. Children were excluded if they have had barium examination or nuclear medicine scan (such as computed tomography and magnetic resonance imaging) that involved an injection of radioactive isotope within the past seven days prior to the DXA examination, as the barium or isotope will affect the accuracy of bone measurements.

### 2.3. Anthropometric Measurements

Body weight was recorded in minimal clothing to the nearest 0.1 kg on a SECA 880 electronic scale (SECA Corp., Hamburg, Germany) and height (without shoes) was measured to the nearest 0.1 cm using a SECA stadiometer Model 213 (SECA Corp., Hamburg, Germany). Measurements were taken twice and the average of the two values for each measurement was used in the data analysis. Body mass index (BMI) was expressed as the ratio of weight to the square of height (kg/m^2^), and BMI-for-age *Z*-scores (BAZ) were calculated using WHO AnthroPlus software version 1.01 (World Health Organization, Geneva, Switzerland).

### 2.4. Radial QUS Measurements

The radial QUS measurements were performed using a commercial device (Omnisense 8000P, Sunlight, Petah Tikva, Israel), which is specifically designed for assessing speed of sound (SOS) (m/s) of ultrasonic waves, which travel axially along the bones at a center frequency of 1.25 MHz using gel as a coupling agent between probe and skin. SOS was measured at distal one-third radius point of the nondominant side of the subject, which was defined as the midpoint of the line between the elbow and the end of the middle finger. The device was calibrated before each data collection session using a verification phantom provided by the manufacturer.

Each subject was scanned twice at the premarked location without repositioning and the average value of the two scans was used in data analysis. SOS *Z*-scores were calculated according to the normative data derived from a sex- and age-matched Asian population, provided by the manufacturer. All the QUS scans were carried out by a single investigator and the intraoperator CV for this population was 2.4%.

### 2.5. DXA Measurements

Bone mineral density (BMD), which is expressed in grams per centimeter squared (g/cm^2^), was measured by DXA technique using the Hologic QDR Series Model Discovery W S/N 84687 (Hologic Inc., Waltham, MA, USA). Compared to other skeletal sites, whole-body BMD scan is recommended for its high reproducibility and excellent precision when assessing total bone mass in children [21]. The instrument was calibrated daily using a spine phantom supplied by the manufacturer before the measurements.

In brief, subjects were required to wear specific clothing provided by the hospital and to remove all metal objects from the body prior to the scan. The subjects were then positioned supine on the scanning table and were instructed to stay motionless throughout the scan. Each complete scan took approximately ten minutes. BMD *Z*-scores were calculated based on the database of normal age- and sex-matched Caucasian population delivered by the DXA manufacturer. All the scans were conducted and analyzed by the same trained technician from Universiti Kebangsaan Malaysia Medical Center according to the standard operational procedures. The temporal machine precision (CV%) for this study was 0.27%.

### 2.6. Statistical Analysis

Data are presented as mean ± standard deviation (SD) for numerical variables and frequency and percentage for categorical variables, unless otherwise stated. Normal distribution of the variables was confirmed using the Kolmogorov-Smirnov test (*n* > 100). *t*-tests were conducted to test differences between the sexes. Cross-classification analysis was used to identify the proportion of subjects who were correctly classified (same or adjacent quartiles) and grossly misclassified (more than one quartile) by radial QUS compared to the DXA measures. Bland-Altman plot analysis [22] was applied to further evaluate the agreement between the two techniques. A receiver operating characteristics analysis was conducted and the area under the curve (AUC) was calculated to evaluate the potential of radial QUS to distinguish subjects with normal and low BMD as diagnosed by DXA (BMD *Z*-scores ≤ −2.0) [6]. The corresponding optimal cut-off value for the parameter of radial QUS for the classification of bone status was defined based on the sensitivity and specificity values obtained from the ROC curve. A* P *value less than 0.05 was considered statistically significant. Statistical analysis was conducted using Statistical Package for Social Sciences (SPSS) version 17.0 for Windows (SPSS Inc., Chicago, IL).

## 3. Results

### 3.1. Demographic Features of the Study Population

The characteristics of the study population are presented in Table 1. The study group consisted of 69 boys and 65 girls with a mean age of 9.4 ± 1.1 years. No significant differences were found between the sexes for the anthropometric and bone parameters. Five subjects (one boy and four girls) had low BMD (*Z*-score ≤ −2.0). Although these five subjects had lower SOS values (3569 ± 57 m/s; *P* < 0.001) compared to subjects with normal BMD status (3702 ± 75 m/s), none of them had an SOS *Z*-score below −2.

### 3.2. Agreement between Radial SOS and Whole-Body BMD

To assess the agreement between the two techniques, subjects were categorized into quartiles of radial SOS and BMD measures (Table 2). Cross-classification analysis showed that 94 (70.1%) subjects were correctly classified (into the same or adjacent quartiles), while 40 (29.9%) of them were grossly misclassified (more than 1 quartile apart) according to the quartiles of BMD for SOS measures. Additional analyses (Table 3) showed that more girls than boys were misclassified but age and BMI did not differ between correct and incorrect classified children.

**Figure 1:**
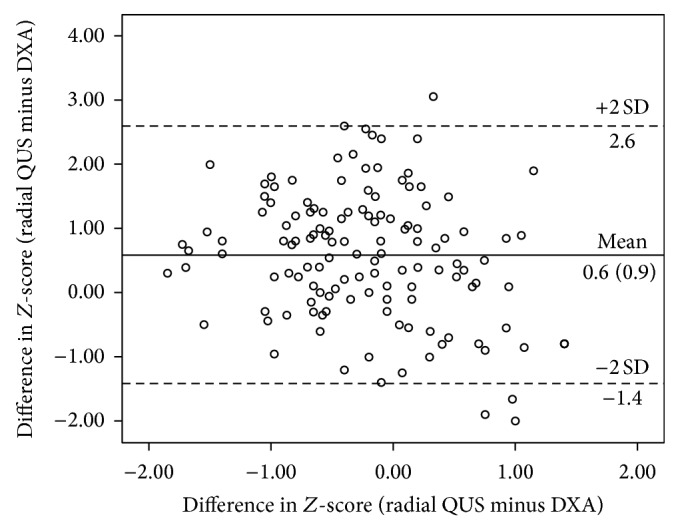
Bland-Altman plot for assessing the agreement between radial quantitative ultrasound (QUS) and dual energy X-ray absorptiometry (DXA).

To further evaluate the agreement between radial QUS and DXA, differences in *Z*-scores between radial SOS and BMD were plotted against the mean of the two techniques using Bland-Altman plot analysis (Figure 1). The mean difference between the two techniques was relatively big (0.6 ± 0.9; *P* < 0.001) and the limits of agreement were large (ranging from −1.4 to 2.6), reflecting considerable amount of discrepancies between the measurements of the two techniques. The bias was negatively correlated with BMI (*r* = −0.41, *P* < 0.001) and positively correlated with age (*r* = 0.17, *P* < 0.05), indicating that SOS *Z*-score is underestimated in children with higher BMI and overestimated in older children when DXA-derived BMD *Z*-scores were referred to as the standard in this context.

### 3.3. Diagnostic Value of Radial SOS

**Figure 2:**
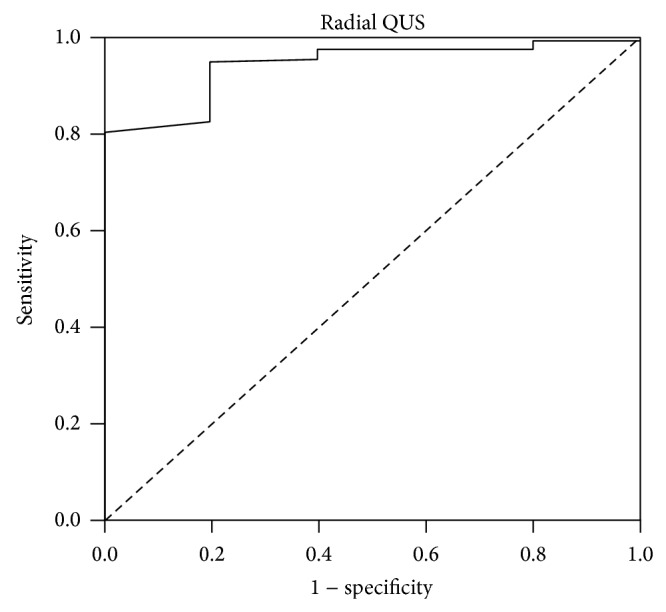
Receiver operating characteristics (ROC) curve for radial speed of sound (SOS) in predicting low bone mineral density (BMD) using whole-body BMD *Z*-scores as the standard.

Using whole-body BMD as the reference to distinguish subjects with normal and low bone mass status, a ROC curve (Figure 2) showed an AUC of 0.94 (95% CI: 0.89, 0.99; *P* < 0.001). Several cut-off values of radial SOS were selected from the ROC curve and their corresponding sensitivities and specificities are shown in Table 4. With a cut-off value of −2.0 or lower, radial SOS yields zero sensitivity (0%) but perfect specificity (100%). However, at a *Z*-score value of −1.0, it shows higher sensitivity (80%) while maintaining a satisfactory specificity (93%).

## 4. Discussion

With rapid technological developments in ultrasound imaging, the use of QUS technique in bone studies has increased significantly over the last decade. However, its validity against conventional radiographic techniques as a measure of bone mass in childhood remains uncertain. In the present study, radial QUS demonstrated only weak correlations with whole-body DXA measures. A high proportion of the children were misclassified in their bone status using radial QUS compared to DXA. The agreement between the two techniques was clinically unsatisfactory and suggests that radial QUS may not be a good alternative for assessing bone status in children when compared to the established standard: DXA.

Several studies have validated radial QUS with DXA at different skeletal regions in pediatric populations and the results varied considerably with correlation coefficients between insignificant and 0.6 [14–16]. It should be noted, however, that the output from bivariate correlation analysis does not indicate whether the two techniques are comparable or having the same relative accuracy. When quartiles of radial SOS were cross-tabulated with quartiles of whole-body BMD measures, nearly 30% of the children were grossly misclassified. Bland and Altman analysis also revealed a significant bias (with radial SOS providing a lower estimation) and more importantly large limits of agreement between the *Z*-scores of measures, indicating that both techniques are not comparable. This is in concordance to the results of a recent study conducted by Williams et al. [17], where the disagreement rate between radial QUS (Omnisense 7000P) and DXA techniques for “abnormal scans” (*Z*-scores ≤ −2) in healthy and diseased populations was relatively high (6–14%).

In contrast to the negative findings as revealed by Bland-Altman plot analysis, ROC analysis provided reasonable results with the AUC of radius SOS approaching 1.0 (0.94), indicating a high discriminatory ability for radial QUS to distinguish children with low and normal BMD status. It should be noted, however, that the proportion of subjects with low BMD in the present study was small (five subjects) and this may limit the ability for an AUC difference analysis to be sufficiently powered in detecting significant differences in values derived from the ROC curves [23]. Also, none of the five children with a BMD *Z*-score < −2 had an SOS *Z*-score < −2. It has to be kept in mind that Bland-Altman plot analysis focuses on the whole range of measured values, which may yield a more valid comparison between the two techniques compared to ROC. In fact, in the study by Khan et al. [14], the predictive ability of radial SOS *Z*-scores for BMD *Z*-scores at lumbar spine site (from ≤ −2 to ≥ 1) was generally poor, with the sensitivity values ranging from 22–53%; although the specificity value was high for those with high (*Z*-score ≥ 1) (left radius: 89%; right radius: 93%) or very low BMD (*Z*-score ≤ −2) (91% for both left and right radius).

The observed relatively high misclassification rate and poor agreement in the present study confirms that the techniques are not interchangeable in assessing bone status of children. There are several possible reasons to explain these discrepancies. First, the regions of interests measured by both techniques are different in this study (regional compared to whole body) and each region comprised different proportions of trabecular and cortical bone that react differently towards the mechanical signals, which arise from the local bone [24]. Hence, the bone status at one skeletal site does not necessarily reflect those at another. Moreover, ultradistal radius is a non-weight-bearing site, which is less regulated by the typical mechanical loading environment compared to the whole-body region that covers the entire skeleton with both weight- and non-weight-bearing bones. Therefore, it should not be surprising to find a relatively poor agreement between the measures of both techniques, since they do not measure identical properties of bone tissues.

Differences may also be attributed to variation in thickness and composition of the soft tissue layers overlying the bone surface, as this can induce significant inaccuracies and measurement errors, thus limiting the predictive ability of the QUS parameters [25, 26]. In fact, it has been reported that QUS scans could not be conducted in many obese children and the disagreement between radial QUS and DXA was higher in obese children (11.5%) compared to normal weight children (7.5%), suggesting that the thickness of the overlying soft tissue does affect the transmission of QUS signals in the obese individuals [17]. This is in accordance with findings of the present study, where the differences in *Z*-scores values of both techniques (QUS minus DXA) were negatively correlated to BMI. Some studies suggest that the effect of soft tissue layer is dependent on the pathways of ultrasound transmission inside the bone, with strong impact being observed in QUS with transverse transmission system (phalangeal and calcaneal) [27, 28] and little or no influence on those with axial transmission system (radial and tibial) [29]. These findings, however, could not be tested in this study as the data on the soft tissues measurements were not available.

Another factor that limits the application of the QUS technique in the general population is the lack of a universal cut-off point for QUS parameters. Although the International Society for Clinical Densitometry (ISCD) has recommended the use of a *Z*-score cutoff of −2 to define low bone mineral content or BMD in children [6], this does not apply equally to techniques other than DXA. This is clearly seen in the present study, whereby none of the subjects with low BMD as determined by DXA were found to have SOS *Z*-scores < −2. Therefore, it seems important to establish QUS-specific thresholds to avoid misinterpretation of results and at the same time to optimize the effectiveness of QUS in the field of bone assessment.

ROC analysis between radial QUS and DXA measurements in this study suggest that a *Z*-score of −1.0 seems to be a more preferred cutoff for radial SOS, where it gives high values for both sensitivity (80%) and specificity (93%), resulting in an acceptable false negative rate (20%) and a much lower false positive rate (7.0%) compared to the current cutoff of −2. This is in line with the mean difference in *Z*-scores of QUS and DXA of 0.6 ± 0.9.

There are several limitations of this study. Firstly, our study did not include DXA measurement of BMD at the corresponding peripheral region to the one measured by the radial QUS, which may provide better agreement. However, our main interest was to ascertain the predictive ability of the peripheral QUS to represent overall bone status; hence, the DXA scan was performed on the whole body. Moreover, it would be difficult to position an active child to accurately measure BMD at the forearm region, and we could not obtain ethical approval for multiple exposures of our subjects to the DXA scan. Secondly, the normative databases used by radial QUS and DXA in this study are derived from different populations (Asian (mainly Korean: personal communication with manufacturer) versus Caucasian) and this might be part of the reason for the discrepancies between the two techniques, given that the variations in bone status are always complicated by the demographic, geographic, and ethnic background of the population of interest. This calls for local normative values for both radial QUS and DXA to further enhance their validity as bone health assessment tools in this region. However, the different normative databases could explain the mean difference in *Z*-score but not the large SD of the difference. Next, the QUS software provided only the SOS values and the broadband ultrasound attenuation (BUA), which is another ultrasound parameter in the QUS system, was not available. It is known that SOS and BUA reflect different properties of bone but the answer of which parameter produces the best evaluations of bone status remains uncertain. Some QUS devices also provide the combined parameters called stiffness index and quantitative ultrasound index; however, the Sunlight Omnisense QUS does not. Moreover, the usefulness of these parameters in children warrants further clinical evaluation and validation [10]. Finally, the results derived in this study may not be generalized to other similar radial QUS devices because of technological diversities and other device-specific factors and the results may not be applicable to children from other regions even if the same instrument is used. As there is mounting evidence to suggest that QUS provides structural information that is related to the quality rather than the density of a bone alone [10], it is worthwhile to understand the factors associated with bone properties as measured by QUS, for example, nutritional and lifestyle variables [30], to further justify the clinical value of this ultrasonographic technique.

## 5. Conclusions

In conclusion, radial QUS and DXA are not comparable and hence are not interchangeable in evaluating bone status of the children. Nonetheless, this does not mean that ultrasonometry at a peripheral site need to be totally disregarded as a method of evaluating the bone status of the children. In the present study, we proposed an optimal cut-off point of radial SOS: *Z*-score of −1.0, which could be used as a reference in future bone status assessment when using a similar radial QUS device among healthy children.

## Figures and Tables

**Table 1:** General characteristics and bone parameters, categorized with regard to gender.

Characteristics	Boys (*n* = 69)			Girls (*n* = 65)		
	Mean	SD	Range	Mean	SD	Range
Age (years)	9.3	1.0	7.3–11.2	9.4	1.2	7.3–11.2
Weight (kg)	30.7	9.2	18.9–57.5	31.7	10.6	17.4–64.5
Height (cm)	130.7	6.8	116.3–143.3	132.5	10.3	112.4–155.4
BMI (kg/m^2^)	17.7	4.1	12.9–29.0	17.6	3.8	12.9–26.7
BAZ	0.38	1.73	−3.10–4.77	0.24	1.48	−2.29–3.37
Radial SOS (m/s)	3700	77	3540–3903	3693	80	3506–3878
Radial SOS *Z*-score	0.05	0.72	−1.5–2.1	0.04	0.77	−1.8–1.8
BMD (g/cm^2^)	0.766	0.048	0.650–0.888	0.751	0.065	0.639–0.914
BMD *Z*-score	−0.55	0.84	−2.1–1.8	−0.53	0.99	−2.5–2.0

BMI: Body mass index; BAZ: BMI-for-age *Z*-score; SOS: speed of sound; BMD: bone mineral density.

**Table 2:** Distribution of subjects by quartiles of bone status according to radial QUS and DXA measures, *n* (%).

DXA quartile	Radial QUS quartile				
	<25th	25th–50th	50th–75th	>75th	Total
<25th	15 (11.2)	8 (6.0)	4 (3.0)	7 (5.2)	34 (25.4)
25th–50th	6 (4.5)	10 (7.5)	8 (6.0)	9 (6.7)	33 (24.6)
50th–75th	8 (6.0)	8 (6.0)	11 (8.2)	7 (5.2)	34 (25.4)
>75th	4 (3.0)	8 (6.0)	11 (8.2)	10 (7.5)	33 (24.6)

Total	33 (24.6)	34 (25.4)	34 (25.4)	33 (24.6)	134 (100.0)

DXA: Dual energy X-ray absorptiometry; QUS: Quantitative ultrasound.

**Table 3:** Comparison of characteristics between groups of classification.

Characteristics	Gross misclassification (*n* = 40)		Correct classification (*n* = 94)		*P* value
	Mean	SD	Mean	SD	
Age (years)	9.3	1.2	9.4	1.1	0.68^a^
BMI (kg/m^2^)	18.2	4.0	17.5	4.0	0.39^a^
Sex					
Male, *n* (%)	13 (18.8)		56 (81.2)		<0.01^b^
Female, *n* (%)	27 (41.5)		38 (58.5)		

BMI: body mass index.

^a^Using independent *t*-test; ^b^using chi-square test.

**Table 4:** Sensitivity and specificity at various *Z*-score cutoffs for radial SOS.

*Z*-score cutoff	Sensitivity (%)	Specificity (%)
−2.0	0	100
−1.5	20	98
−1.0	80	93
−0.5	100	80
0.0	100	48
0.5	100	32

## References

[1] Johnell O., Kanis J. A. (2006). An estimate of the worldwide prevalence and disability associated with osteoporotic fractures. *Osteoporosis International*.

[2] Shuler F. D., Conjeski J., Kendall D., Salava J. (2012). Understanding the burden of osteoporosis and use of the World Health Organization FRAX. *Orthopedics*.

[3] Bonjour J. P., Chevalley T., Ferrari S., Rizzoli R. (2009). The importance and relevance of peak bone mass in the prevalence of osteoporosis. *Salud Publica de Mexico*.

[4] Bachrach L. K., Sills I. N. (2011). Bone densitometry in children and adolescents. *Pediatrics*.

[5] Specker B. L., Schoenau E. (2005). Quantitative bone analysis in children: current methods and recommendations. *The Journal of Pediatrics*.

[6] Gordon C. M., Bachrach L. K., Carpenter T. O. (2008). Dual energy X-ray absorptiometry interpretation and reporting in children and adolescents: the 2007 ISCD pediatric official positions. *Journal of Clinical Densitometry*.

[7] Goh S. Y., Aragon J. M., Lee Y. S., Loke K. Y. (2011). Normative data for quantitative calcaneal ultrasound in Asian children. *Annals of the Academy of Medicine Singapore*.

[8] International Osteoporosis Foundation (2009). *The Asian Audit. Epidemiology, Costs and Burden of Osteoporosis in Asia 2009*.

[9] Guglielmi G., de Terlizzi F. (2009). Quantitative ultrasound in the assessment of osteoporosis. *European Journal of Radiology*.

[10] Baroncelli G. I. (2008). Quantitative ultrasound methods to assess bone mineral status in children: technical characteristics, performance, and clinical application. *Pediatric Research*.

[11] Krieg M.-A., Barkmann R., Gonnelli S. (2008). Quantitative ultrasound in the management of osteoporosis: the 2007 ISCD official positions. *Journal of Clinical Densitometry*.

[12] Hans D., Krieg M. A. (2009). Quantitative ultrasound for the detection and management of osteoporosis. *Salud Publica de Mexico*.

[13] Binkley T. L., Berry R., Specker B. L. (2008). Methods for measurement of pediatric bone. *Reviews in Endocrine and Metabolic Disorders*.

[14] Khan K. M., Sarafoglou K., Somani A., Frohnert B., Miller B. S. (2013). Can ultrasound be used to estimate bone mineral density in children with growth problems?. *Acta Paediatrica*.

[15] Christoforidis A., Printza N., Gkogka C. (2011). Comparative study of quantitative ultrasonography and dual-energy X-ray absorptiometry for evaluating renal osteodystrophy in children with chronic kidney disease. *Journal of Bone and Mineral Metabolism*.

[16] Jones G., Boon P. (2008). Which bone mass measures discriminate adolescents who have fractured from those who have not?. *Osteoporosis International*.

[17] Williams J. E., Wilson C. M., Biassoni L., Suri R., Fewtrell M. S. (2012). Dual energy x-ray absorptiometry and quantitative ultrasound are not interchangeable in diagnosing abnormal bones. *Archives of Disease in Childhood*.

[18] Poh B. K., Ng B. K., Siti Haslinda M. D. (2013). Nutritional Status and dietary intakes of children aged 6 months to 12 years: findings of the Nutrition Survey of Malaysian Children (SEANUTS Malaysia). *British Journal of Nutrition*.

[19] Schaafsma A., Deurenberg P., Calame W. (2013). Design of the South East Asian Nutrition Survey (SEANUTS): a four-country multistage cluster design study. *British Journal of Nutrition*.

[20] Faul F., Erdfelder E., Buchner A., Lang A.-G. (2009). Statistical power analyses using G^∗^Power 3.1: tests for correlation and regression analyses. *Behavior Research Methods*.

[21] Baim S., Leonard M. B., Bianchi M.-L. (2008). Official positions of the international society for clinical densitometry and executive summary of the 2007 ISCD pediatric position development conference. *Journal of Clinical Densitometry*.

[22] Bland J. M., Altman D. G. (1986). Statistical methods for assessing agreement between two methods of clinical measurement. *The Lancet*.

[23] Obuchowski N. A. (2000). Sample size tables for receiver operating characteristic studies. *The American Journal of Roentgenology*.

[24] Brandi M. L. (2009). Microarchitecture, the key to bone quality. *Rheumatology*.

[25] Malo M. K. H., Karjalainen J. P., Isaksson H., Riekkinen O., Jurvelin J. S., Töyräs J. (2010). Numerical analysis of uncertainties in dual frequency bone ultrasound technique. *Ultrasound in Medicine and Biology*.

[26] Stein E. M., Rosete F., Young P. (2013). Clinical assessment of the 1/3 radius using a new desktop ultrasonic bone densitometer. *Ultrasound in Medicine and Biology*.

[27] Rico H., Gómez M., Aguado F., Villa L. F., Hernández E. R., Cortés J. (1999). Impact of weight in obese subjects on bone speed of sound. *Investigative Radiology*.

[28] Chappard C., Camus E., Lefebvre F. (2000). Evaluation of error bounds on calcaneal speed of sound caused by surrounding soft tissue. *Journal of Clinical Densitometry*.

[29] Steinschneider M., Hagag P., Rapoport M. J., Weiss M. (2003). Discordant effect of body mass index on bone mineral density and speed of sound. *BMC Musculoskeletal Disorders*.

[30] Micklesfield L. K., Zielonka E. A., Charlton K. E., Katzenellenbogen L., Harkins J., Lambert E. V. (2004). Ultrasound bone measurements in pre-adolescent girls: interaction between ethnicity and lifestyle factors. *Acta Paediatrica*.

